# Regulation of *dndB* Gene Expression in *Streptomyces lividans*

**DOI:** 10.3389/fmicb.2018.02387

**Published:** 2018-10-08

**Authors:** Daofeng Dai, Tianning Pu, Jingdan Liang, Zhijun Wang, Aifa Tang

**Affiliations:** ^1^Health Science Center, The First Affiliated Hospital of Shenzhen University, and Institute of Translational Medicine, Shenzhen Second People’s Hospital, Shenzhen, China; ^2^State Key Laboratory of Microbial Metabolism, School of Life Sciences and Biotechnology, Shanghai Jiao Tong University, Shanghai, China

**Keywords:** DNA sulfur modification, epigenetic regulation, transcriptional regulation, *Streptomyces lividans*, *dndB* gene

## Abstract

DNA sulfur modification is a unique modification occurring in the sugar-phosphate backbone of DNA, with a nonbridging oxygen atom substituted with sulfur in a sequence-specific and *R*p stereo-specific manner. Bioinformatics, RNA-seq, and *in vitro* transcriptional analyses have shown that DNA sulfur modification may be involved in epigenetic regulation. However, the *in vivo* evidence supporting this assertion is not convincing. Here, we aimed to characterize two sulfur-modified sites near the *dndB* promoter region in *Streptomyces lividans*. Single mutation of either site had no effect on *dndB* transcription, whereas double mutation of both sites significantly elevated *dndB* expression. These findings suggested that DNA sulfur modification affected gene expression, and the role of DNA sulfur modification in epigenetic regulation depended on the number of sulfur-modified sites. We also identified an inverted repeat, the R repeat sequence, and showed that this sequence participated in the positive regulation of *dndB* gene expression.

## Introduction

DNA sulfur modification, in which a nonbridging oxygen atom in the sugar-phosphate backbone is replaced by a sulfur atom (Wang et al., 2007), is widespread across bacteria and archaea (He et al., 2007; Ou et al., 2009). Sulfur modification is governed by the five-gene cluster *dndABCDE* (Zhou et al., 2005), which was first identified in *Streptomyces lividans*. To date, the *dnd* gene cluster has been detected in 1349 bacterial and archaeal strains (Tong et al., 2018). *dndBCDE* is cotranscribed (Xu et al., 2009), and *dndA* and *dndCDE* are essential for DNA sulfur modification, because deleting *dndA, dndC, dndD*, or *dndE* abolishes DNA sulfur modification (Xu et al., 2009). DndA protein has L-cysteine desulfurase activity and assembles the 4Fe-4S cluster of DndC (You et al., 2007). Additionally, DndB is a negative regulatory protein that binds to the *dndB* promoter region (Cheng et al., 2015; He et al., 2015). DndC possesses ATP pyrophosphatase activity and has high sequence homology with phosphoadenosine phosphosulfate reductase. DndD acts as an ATPase and may be required for introducing nicks in DNA during sulfur incorporation (Yao et al., 2009). DndE is a tetramer sensitive to nicked double-stranded DNA (Hu et al., 2012).

DNA sulfur modification occurs in an *R*_p_ stereo-specific (Wang et al., 2007), sequence-selective (Dyson and Evans, 1998; Liang et al., 2007; Wang et al., 2011), low-frequency manner (Cao et al., 2014a). In *S. lividans* 1326, sulfur-modified d(GpsG), at a frequency of 471 ± 39 per 10^6^ nt, has been identified within the conserved sequence GGCC (Wang et al., 2011); in *Escherichia coli* B7A, sulfur-modified d(GpsA) and d(GpsT) located within the consensus sequences GAAC and GTTC have been identified at frequencies of 370 ± 11 and 398 ± 17 per 10^6^ nt, respectively (Wang et al., 2011); and in *Vibrio cyclitrophicus* FF75, sulfur-modified d(CpsC), at a frequency of 2624 ± 22 per 10^6^ nt, has been identified within the conserved motif CCA (Wang et al., 2011). Incomplete modification is observed for *E. coli* B7A and *V. cyclitrophicus* FF75. Only 12% of GAAC/GTTC sites on the genome are sulfur-modified in *E. coli* B7A, and 14% of CCA sites on the chromosome are sulfur-modified in *V. cyclitrophicus* FF75 (Cao et al., 2014a).

DNA sulfur modification has been found to be a restriction-modification system, in which the products of *dndABCDE* genes are responsible for the modification, and the products of *dndFGH* genes function as the restriction component to recognize and cleave foreign DNA (Xu et al., 2010; Cao et al., 2014b; Gan et al., 2014). Moreover, 54.4% of 1349 identified *dnd* gene clusters possess both the modification component and the restriction component (Tong et al., 2018). Without DNA sulfur modification, DNA is vulnerable to attack from the restriction component. Thus, the restriction-modification system can protect the strain from foreign infection, such as phage infection.

We previously identified DNA sulfur modification as an antioxidant system, in which sulfur-modified DNA scavenged different types of peroxides, such as hydrogen peroxide (Xie et al., 2012), organic hydroperoxide (Dai et al., 2016), and hydroxy radical (Yang et al., 2017), and strains possessing DNA sulfur modification were resistant to peroxide treatment.

Methylated DNA can affect the helicity of DNA, indicating the association between DNA helicity and function (Vargason et al., 2000, 2001). X-ray crystallography analyses have suggested that there are three basic types of helices, namely, A-, B-, and Z-helix (Saenger et al., 1986; Schneider et al., 1997; Svozil et al., 2008). B-helix, which are the most prevalent in the DNA double-helix structure, are destabilized by DNA sulfur modification (Zhang et al., 2012; Chen et al., 2015). Thus, DNA sulfur modification may be involved in epigenetic regulation. Indeed, RNA-seq analyses and *in vitro* transcriptional analyses by Tong et al. revealed that DNA sulfur modification could affect epigenetic control (Tong et al., 2018).

DNA sulfur modification is partial at genomic sites in *E. coli* B7A and *V. cyclitrophicus* FF75 (Cao et al., 2014a), indicating regulation of the level of DNA sulfur modification. Indeed, in *Salmonella enterica*, DndB protein negatively regulates the transcription of *dndBCDE* by binding to two direct repeats in the *dndB* promoter region, each containing the sequence TACGN(10)CGTA (He et al., 2015).

In this study, we evaluated the correlation between sulfur-modified sites and gene regulation *in vivo* by analyzing the effects of sulfur modification near the *dndB* promoter region on *dndB* gene expression. Our results suggested that DNA sulfur modification was involved in epigenetic regulation. Moreover, we also identified an inverted repeat sequence, the R repeat sequence, and showed that this sequence participates in the positive regulation of *dndB* gene regulation.

## Materials and Methods

### Bacterial Strains, Plasmids, and Primers

Bacterial strains, plasmids used in this study are listed in **Supplementary Table S1**. *E. coli* strains were cultured in Luria-Bertani medium at 37°C. SFM agar medium was used for sporulation of *Streptomyces* strains and for conjugation between *E. coli* ET12567/pUZ8002 and *Streptomyces* (Kieser et al., 2000). For isolation of DNA or RNA from *Streptomyces*, the rich liquid medium 34% TSBY (30 g/L tryptic soy broth, 5 g/L yeast, 340 g/L sucrose) was used. *Streptomyces* strains were grown at 30°C. Ampicilin and apramycin were added to the medium at final concentrations of 100 and 30 μg/mL, respectively.

Primers used in this study are listed in **Supplementary Table S2**.

### Mutagenesis of DNA Sequence and the Assay of Catechol 2,3-Dioxygenase Activity

DNA sequence was mutated according to the following procedures. First, a high-fidelity PCR reaction was performed in a mixture of 50 μL containing 50 ng of template DNA, 1x HF buffer, 50 μM dNTP, 6% DMSO, 1 μM primer pair, 1 unit of Phusion DNA Polymerase (Thermo). To remove methylated template DNA, PCR product was digest using DpnI at 37°C overnight, in a reaction volume of 50 μL containing 44 μL of PCR product, 1x Tango buffer, 10 units of DpnI (Thermo). Then, T4 Polynucleotide Kinase (NEB) was used to add phosphate to 5′ terminus of PCR product. The reaction mixture consisting of 17 μL of DpnI-treated PCR product, 1x T4 DNA ligase buffer, and 10 units of T4 Polynucleotide Kinase, was incubated at 37°C for 30 min, followed by heat inactivation at 65°C for 20 min. The phosphorylated PCR product was self-ligated using Solution I (Takara). A reaction volume of 20 μL containing 10 μL of phosphorylated PCR product and 10 μL of Solution I was incubated at 16°C for 2 h. The ligation mixture was directly transformed into *E. coli* DH5α.

The assay of catechol 2,3-dioxygenase activity was performed as described without modification (Ingram et al., 1989).

### Determination of DNA Sulfur-Modified Sites

To determine sulfur-modified sites near *dndB* promoter region, genomic DNA from *S. lividan* wild-type or mutant strains was sequentially treated with restriction endonuclease and PAA. Treatment with PAA of DNA was conducted based on the protocol of Xie et al. (2012). Then treated DNA was separated by running agarose gel electrophoresis. Finally, Southern blotting was performed to determine DNA sulfur-modified sites. Southern blotting was conducted according to manufacturer’s instructions (Roche).

### Quantitative RT-PCR

RNA was isolated from *S. lividans* strains, and RNA concentration was measured using the NanoDrop 2000 Spectrophotometer (Thermo). Genomic DNA was removed using RNase-free DNase I, and elimination of DNA from RNA was further confirmed using PCR. Reverse transcription was conducted with the cDNA Synthesis Kit (Thermo), and the resulting cDNA was used as the template for quantitative real-time PCR. The expression of *dndC, dndD*, and *dndE* was quantitatively analyzed; *rrnA* encoding 16s rRNA was used as the internal control. Forward and reverse primers are listed in **Supplementary Table S2**. PCR products were examined by electrophoresis on a 1.2% agarose gel and visualized by staining with ethidium bromide.

For quantitative real-time PCR, reactions were performed on an ABI7500 Fast Real Time System (Applied Biosystems). A reaction mixture of 25 μL contained 1 or 150 ng of cDNA (1 ng for reference gene, 150 ng for target gene), 50 nM forward and reverse primers, and 12.5 μL SYBR Green qPCR Master Mix (Thermo). The conditions for PCR amplification were 95°C for 10 min, followed by 40 cycles of 95°C for 15 s, 60°C for 1 min. A dissociation curve ranging from 55 to 90°C in 0.5°C increments, with a dwell time of 30 s, was performed to assess the specificity of the reactions.

### Quantification of Sulfur-Modified Sites in Genomic DNA

DNA sulfur modification level was analyzed according to the protocol of Cao et al. (2014a). Briefly, 50 μg of genomic DNA in 89 μL of water was digested with 1 unit of P1 nuclease (Sigma) in 1x nuclease P1 buffer (30 mM NaAc pH 5.3, 5 mM ZnCl2, 50 mM NaCl) at 37°C overnight. Then 1 unit of FastAP (Thermo) and 1x FAST AP buffer were added to the mixture and incubated further at 37°C for 4 h. The tubes were then boiled for 10 min and centrifuged at 18,000 g for 20 min at 4°C. The reaction supernatant was loaded into an Amicon Ultra 0.5 mL Centrifugal Filter (Merck) and centrifuged at 8000 g for 2 h at 4°C. Twenty microliter of the filtrate were loaded onto an Agilent-C18 reverse-phase column (250 mm × 4.6 mm, 5 μm), fitted to an Agilent 1290-MS 6230 HPLC. The solvents were solvent A: 0.1% acetic acid in water and solvent B: 0.1% acetic acid in acetonitrile. The flow rate was 0.4 mL/min and the temperature was 30°C. Gradient conditions were 1–13% solvent B for 10 min, 13–30% solvent B for 20 min and 1% solvent B for 10 min. Ionization was set in positive mode, gas flow was 10 L/min, nebulizer pressure was 30 psi, drying gas temperature was 325°C and capillary voltage 3100 V. Dinucleotide d(G_PS_A) was monitored at m/z 597.1388 whilst d(G_PS_T) at m/z 588.1272.

## Results

### Determination of DNA Sulfur-Modified Sites Near the *dndB* Promoter

**FIGURE 1:**
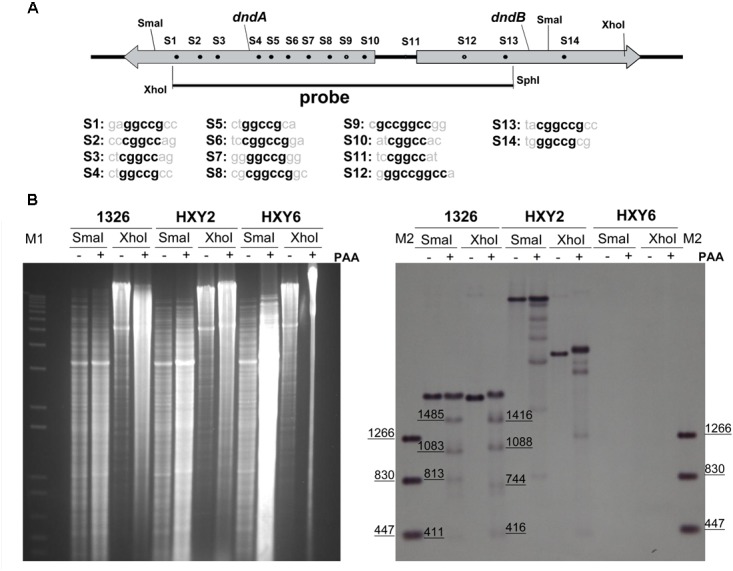
Characterization of DNA sulfur-modified sites near *dndB* promoter region. **(A)** Potential DNA sulfur-modified sites near *dndB* promoter region. Sequences of 14 putative DNA sulfur-modified sites are aligned, and the consensus contexts are in bold. **(B)** To characterize DNA sulfur-modified sites near *dndB* promoter region, Genomic DNA from 1326, HXY2, and HXY6, was digested by *Sma*I or *Xho*I, then treated with PAA, separated on 0.7% agarose gel, and finally characterized using Southern blotting. “-” indicates without PAA; “+” indicates with PAA added. DNA markers for agarose gel electrophoresis are labeled as “M1”; DNA markers for Southern blotting are labeled as “M2.”

To study the effects of DNA sulfur-modified sites near the *dndB* promoter on *dndB* gene transcription, we first identified DNA sulfur-modified sites near the *dndB* promoter. Because DNA sulfur modification in *S. lividans* occurs in the conserved sequence CGGCC or GGCCG (Liang et al., 2007), the fragment containing the *dndA* gene, *dndB* gene, and intergenic region harbored a total of 14 possible DNA sulfur-modified sites (**Figure 1A**).

The strategy to identify DNA sulfur-modified sites was as follows. First, genomic DNA from strains 1326, HXY2, and HXY6 were digested with restriction endoenzymes, such as *Xho*I or *Sma*I. Strain HXY2 is a *dndB* gene in-frame deletion mutant while HXY6 is a *dndABCDE* gene cluster deletion mutant; both strains were derived from *S. lividans* 1326. DNA was then treated with peracetic acid (PAA) to specifically cleave genomic DNA at DNA sulfur-modified sites, and separated on agarose gels in TAE buffer plus thiourea. Finally, Southern blotting was performed.

As shown in **Figure 1B**, the sample from strain HXY6 did not produce any bands, indicating that the probe used in hybridization was very specific. DNA from strain 1326 produced one band if digested with *Xho*I or *Sma*I; PAA treatment following digestion with the restriction endoenzyme produced four smaller degradation bands. These results suggested that the fragment containing the *dndA* gene, *dndB* gene, and intergenic region harbored two DNA sulfur-modified sites. Based on the sizes of these small degraded bands, we speculated that the possible DNA sulfur-modified sites in this fragment were S9 and S12, which are located at nt positions -343 and +329 relative to the transcription start site of the *dndB* gene, respectively.

**FIGURE 2:**
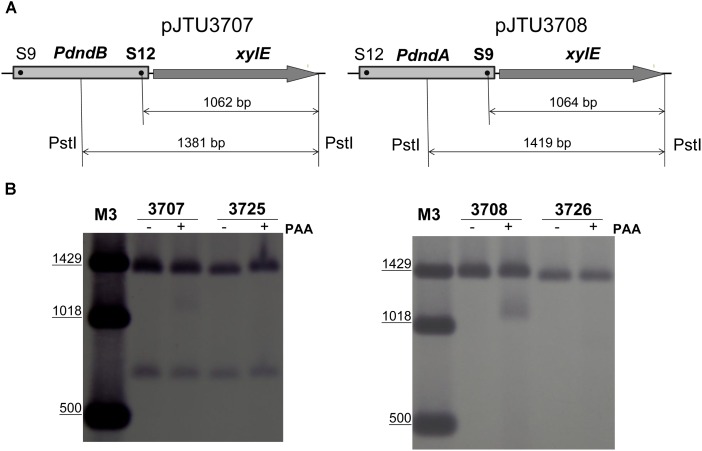
Validation of S9 and S12 sulfur-modified sites. **(A)** Validation scheme for the S9 and S12 sulfur-modified sites. Genomic DNA samples from 1326/pJTU3707, 1326/pJTU3725, 1326/pJTU3708, and 1326/pJTU3726 were, respectively, subjected to digestion with *Pst*I, followed by treatment with PAA. pJTU3707, *PdndB-xylE* reporter plasmid; pJTU3725, pJTU3707 derivative with mutation in S12 site; pJTU3708, *PdndA-xylE* reporter plasmid; pJTU3726, pJTU3708 derivative with mutation in S9 site. **(B)** The effect of mutation in S9 or S12 site on DNA degradation. After digestion with *Pst*I and treatment with PAA, DNA was separated on 0.7% agarose gel, and Southern blotting was performed. “+” indicates PAA treatment; “-” indicates no PAA treatment. DNA markers for Southern blotting are labeled as “M3.”

To verify that S9 and S12 sites were sulfur-modified, we mutated the S12 site in pJTU3707 and the S9 site in pJTU3708 (**Figure 2A**), producing pJTU3725 and pJTU3726, respectively. pJTU3707 was derived from pSET152 and carried the *PdndB*-*xylE* construct. The direction of the *dndB* promoter in pJTU3708 was opposite to that in pJTU3707. Then, pJTU3707, pJTU3708, pJTU3725, and pJTU3726 were transferred into *S. lividans* 1326 through conjugation. Genomic DNA from the resulting *S. lividans* strains was digested with *Pst*I, and sequentially subjected to PAA treatment, separation by agarose gel electrophoresis, and Southern blotting. As shown in **Figure 2B**, 1326/pJTU3707 and 1326/pJTU3708 both produced a degraded band at around 1 kb, and mutation of S9 and S12 sites eliminated the degraded band, respectively. These results suggested that the S9 and S12 sites were sulfur-modified.

### Effects of Sulfur-Modified Sites on *dndB* Gene Expression

**FIGURE 3:**
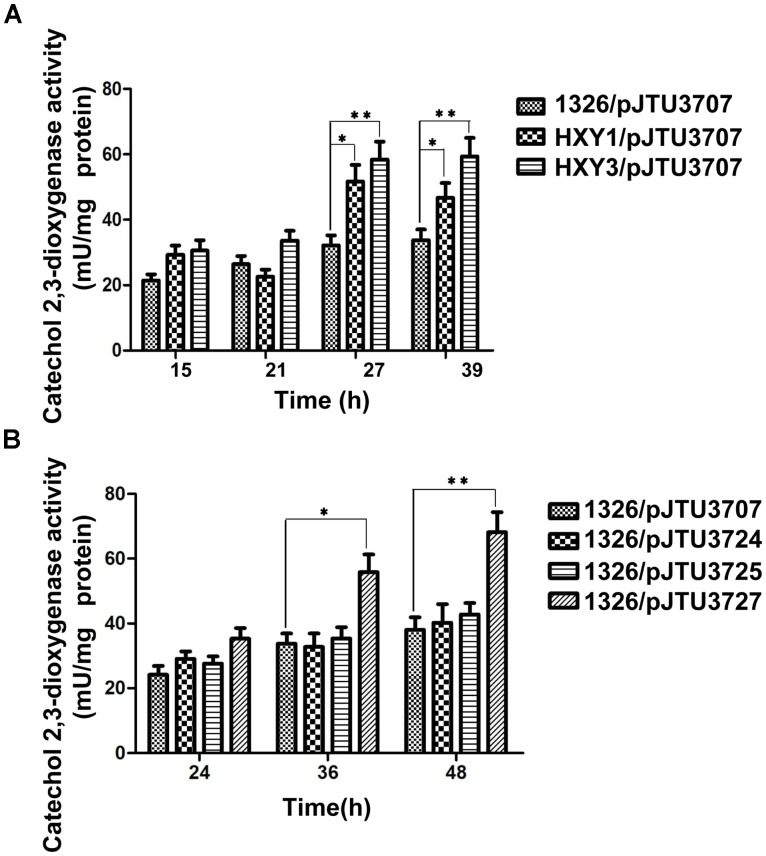
The effects of S9 and S12 sulfur-modified sites on *dndB* transcription**. (A)** Histogram showing assays of quantitative catechol 2,3-dioxygenase activity from 1326/pJTU3707, HXY1/pJTU3707, and HXY3/pJTU3707. HXY1, 1326 derivative with *dndA* deleted; HXY3, 1326 derivative with *dndC* deleted. **(B)** Histogram showing assays of quantitative catechol 2,3-dioxygenase activity from 1326/pJTU3707, 1326/pJTU3724, 1326/pJTU3725, and 1326/pJTU3727. pJTU3724, pJTU3707 derivative with single mutation in S9 site; pJTU3727, pJTU3707 derivative with double mutations in S9 and S12 sites. ^∗^*P* < 0.05; ^∗∗^*P* < 0.01. Three replicates were performed.

To test whether DNA sulfur modification affected gene expression, pJTU3707 was transferred into strains HXY1 and HXY3 through conjugation. Strain HXY1 harbored a *dndA* gene deletion mutation, and HXY3 harbored a *dndC* gene deletion mutation. Both strains were derived from *S. lividans* 1326. Because *dndA* and *dndC* are both required for DNA sulfur modification, strains HXY1 and HXY3 did not possess DNA sulfur modification activity. A comparison of the catechol 2,3-dioxygenase activities of 1326/pJTU3707, HXY1/pJTU3707, and HXY3/pJTU3707 showed that transcription of the *dndB* gene increased about 60–80% upon loss of DNA sulfur modification (**Figure 3A**). These findings suggested that DNA sulfur modification may repress the expression of the *dndB* gene.

**FIGURE 4:**
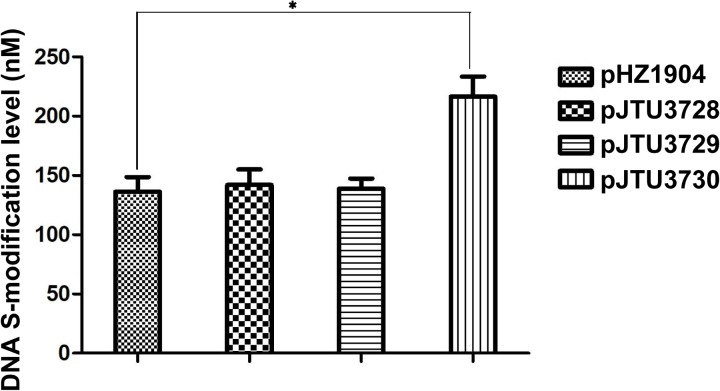
The effect of mutation in S9 and S12 sulfur-modified sites on DNA sulfur modification level. pHZ1904 and its derivatives were transferred into HXY6 through conjugation, and DNA sulfur modification abundance for each strain was compared. pJTU3728, pHZ1904 derivative with single mutation in S9 site; pJTU3729, pHZ1904 derivative with single mutation in S12 site; pJTU3730, pHZ1904 derivative with double mutations in S9 and S12 sites. ^∗^*P* < 0.05. Three replicates were performed.

To further confirm these findings, we mutated the S9 and S12 sites to test the effects of mutation on *dndB* gene expression. As shown in **Figure 3B**, a comparison of catechol 2,3-dioxygenase activities showed that single mutation of the S9 or S12 site had no apparent effect on *dndB* gene transcription. However, double mutation of both sites caused a significant increase in *dndB* gene expression. We mutated the S9 and S12 sites on pHZ1904 to test the effects of mutation on the phenotype. Single mutation of the S9 or S12 site had no effect on DNA sulfur modification abundance; in contrast, double mutation of both sites significantly elevated DNA sulfur modification levels (**Figure 4**). These results confirmed that DNA sulfur-modified sites near the *dndB* promoter affected *dndB* gene expression, but in a manner dependent on the number of DNA sulfur-modified sites.

### Identification of the Positive Regulatory Sequence of *dndB* Gene Transcription

**FIGURE 5:**
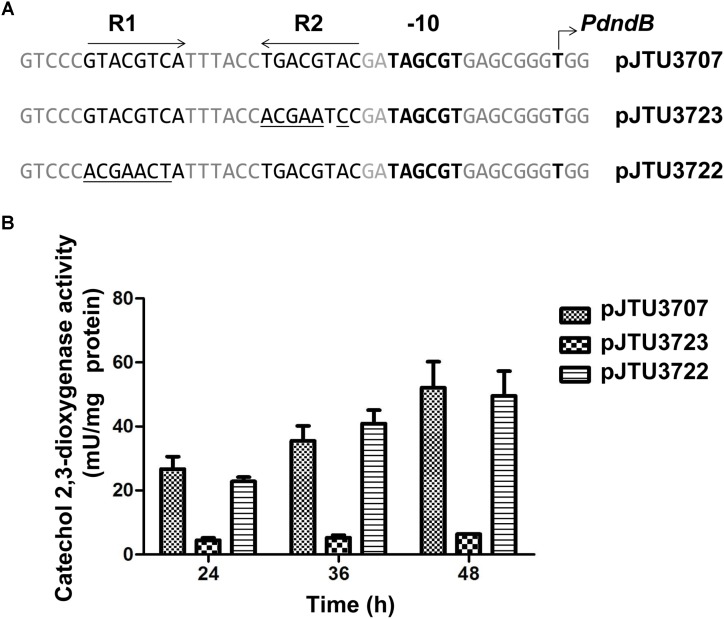
Analysis of mutations within R1 or R2 in *dndB* promoter region. **(A)** Alignment of native *dndB* promoter with its derivatives possessing base substitutions within R1 or R2 (the base substitutions in the derivatives are underlined). **(B)** Histogram showing results of quantitative catechol 2,3-dioxygenase assays from *S. lividans* 1326 containing pJTU3707, pJTU3722, or pJTU3723 **(A)**. At indicated time, samples of each culture were removed to assay catechol 2,3-dioxygenase activity. Three replicates were performed.

In our previous work, we identified the transcription initiation site and -10 region of the *dndB* gene in *S. lividans* 1326. Our results showed that the inverted repeat sequence (R repeat sequence) consisting of R1 and R2 was located near the -10 region of the *dndB* gene, and was a potential regulatory sequence. R1 and R2 in pJTU3707 were mutated, and the activity of catechol 2,3-dioxygenase was compared. We found that mutation of R1 did not affect the activity of the *dndB* promoter. However, the transcriptional activity of the *dndB* promoter was almost absent after R2 mutation (**Figure 5**). These findings suggested that the R repeat sequence was a positive regulatory sequence for the *dndB* gene and that R2 played a key role.

**FIGURE 6:**
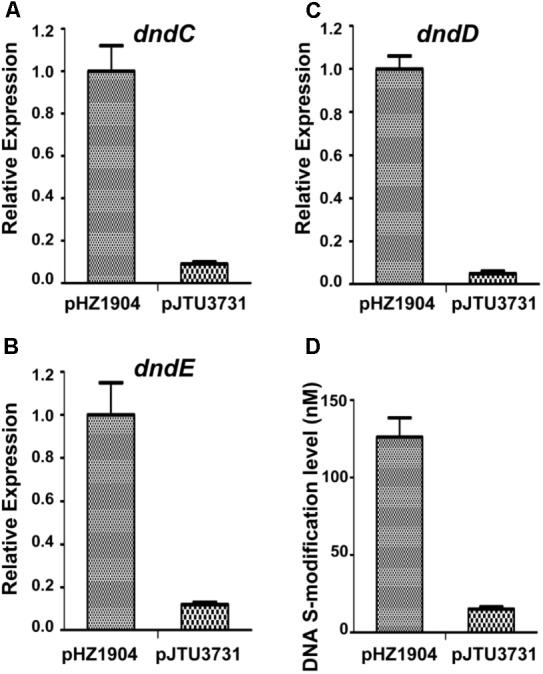
The effects of R2 mutation on *dnd* gene expression and on DNA sulfur modification level. **(A)** The effect of R2 mutation on *dndC* gene expression. **(B)** The effect of R2 mutation on *dndD* gene expression. **(C)** The effect of R2 mutation on *dndE* gene expression. **(D)** The effect of R2 mutation on DNA sulfur modification level. pJTU3731, pHZ1904 derivative with R2 mutation. Three replicates were performed.

Because the *dndBCDE* genes constitute an operon, R2 may affect the transcription of the *dndCDE* genes to alter the abundance of DNA sulfur modification. To verify this hypothesis, the R2 sequence in plasmid pHZ1904 carrying the *dndABCDE* gene cluster was mutated, producing pJTU3731. Next, pHZ1904 and pJTU3731 were transferred into strain HXY6 through conjugation. Strain HXY6 was the *dndABCDE* gene cluster deletion mutant of the wild-type strain *S. lividans* 1326. Mutation of R2 resulted in decreased transcription of *dndC* (**Figure 6A**), *dndD* (**Figure 6B**), and *dndE* (**Figure 6C**). Mutation of R2 also caused a eightfold decrease in the level of DNA sulfur modification (**Figure 6D**). These results suggested that the R repeat sequence consisting of R1 and R2 positively regulated the expression of *dndBCDE* genes to affect the level of DNA sulfur modification.

## Discussion

Several studies have confirmed that DNA sulfur modification is a bacterial restriction-modification system. However, nearly half of the 1349 identified *dnd* gene clusters only possess the modification function and lack the restriction function (Tong et al., 2018). Thus, for strains lacking the restriction function, the role DNA sulfur modification plays is still unclear. In this study, we compared *dndB* gene expression between the *S. lividans* wild-type strain and *dnd* gene deletion mutant strains and analyzed the effects of mutating DNA sulfur-modified sites on *dndB* gene transcription. Our results clearly confirmed that DNA sulfur-modified sites regulate gene expression.

DNA sulfur modification is incomplete at genomic sites. For example, only 12% of GAAC/GTTC sites on the genome are sulfur-modified in *E. coli* B7A (Cao et al., 2014a). Here, we demonstrated that 2 of 14 putative sulfur-modified sites were modified. Importantly, sulfur modification did not randomly occur in the 14 putative sites, but constantly occurred in the S9 and S12 sites, which indicated that the sequence of the sulfur-modified site may be different from that of the unmodified site. Indeed, the S9 and S12 sites both harbored the sequence GCCGGCC, which is different from the sequences of the 12 unmodified sites (**Figure 1A**). It suggested that GCCGGCC may be the conserved context of sulfur-modified sites in *S. lividans*. The conserved sequence of the sulfur-modified site may recruit transcriptional regulators to form complexes, thereby interfering with the function of RNA polymerase, and eventually repressing gene expression.

Our study showed that single mutation of the S9 or S12 site had no significant effect on *dndB* gene transcription, whereas double mutations in S9 and S12 sites affected *dndB* gene expression. These findings suggested that repression by DNA sulfur modification may rely on the number of sulfur-modified sequences. More sulfur-modified sequences may strengthen the interactions between some proteins and the sulfur-modified sites, thereby forming tight complexes and eventually elevating the repression of gene expression.

When analyzing the *cis*-acting elements affecting *dndB* gene expression, we identified an inverted repeat sequence, the R repeat sequence, which was involved in positive regulation of *dndB* gene transcription. Regulation of the *dndBCDE* gene cluster in *Salmonella* has been reported. In *Salmonella*, DndB protein binds to the promoter region of the *dndB* gene to inhibit the expression of the *dndBCDE* gene cluster (Cheng et al., 2015; He et al., 2015). Because the R repeat sequence in *S. lividans* is a positive regulatory sequence, the mechanism regulating *dndB* gene expression in *S. lividans* is different from that in *Salmonella*. DndB protein in *S. lividans* is a negative regulator of *dndB* gene expression. Thus, the protein binding to the R repeat sequence may not be DndB protein, and DNA affinity assays should be used to identify the protein binding to the R repeat sequence.

In summary, our results showed that DNA sulfur-modified sites near the *dndB* promoter region repressed *dndB* gene expression in a manner dependent on the number of sulfur-modified sites. The inverted repeat sequence (the R repeat sequence) was identified as a positive regulatory sequence of *dndB* gene expression.

## Author Contributions

DD and TP did the experiments. DD, TP, JL, ZW, and AT analyzed the data. DD wrote the manuscript text. DD, JL, ZW, and AT prepared figures. All authors reviewed the manuscript.

## Conflict of Interest Statement

The authors declare that the research was conducted in the absence of any commercial or financial relationships that could be construed as a potential conflict of interest.

## References

[B1] Cao B. Chen C. DeMott M. S. Cheng Q. Clark T. A. Xiong X. (2014a). Genomic mapping of phosphorothioates reveals partial modification of short consensus sequences. *Nat. Commun.* 5:3951. 10.1038/ncomms4951 24899568 PMC4322921

[B2] Cao B. Cheng Q. Gu C. Yao F. DeMott M. S. Zheng X. (2014b). Pathological phenotypes and *in vivo* DNA cleavage by unrestrained activity of a phosphorothioate-based restriction system in *Salmonella*. *Mol. Microbiol.* 93 776–785. 10.1111/mmi.12692 25040300 PMC4414249

[B3] Chen L. Wang X.-L. Shi T. Wu T. Deng Z. Zhao Y.-L. (2015). Theoretical study on the relationship between rp-phosphorothioation and base-step in S-DNA: based on energetic and structural analysis. *J. Phys. Chem. B* 119 474–481. 10.1021/jp511359e 25519472

[B4] Cheng Q. Cao B. Yao F. Li J. Deng Z. You D. (2015). Regulation of DNA phosphorothioate modifications by the transcriptional regulator DptB in *Salmonella*. *Mol. Microbiol.* 97 1186–1194. 10.1111/mmi.13096 26096787

[B5] Dai D. Du A. Xiong K. Pu T. Zhou X. Deng Z. (2016). DNA phosphorothioate modification plays a role in peroxides resistance in *Streptomyces* *lividans*. *Front. Microbiol.* 7:1380. 10.3389/fmicb.2016.01380 27630631 PMC5005934

[B6] Dyson P. Evans M. (1998). Novel post-replicative DNA modification in *Streptomyces*: analysis of the preferred modification site of plasmid pIJ101. *Nucleic Acids Res.* 26 1248–1253. 10.1093/nar/26.5.1248 9469833 PMC147391

[B7] Gan R. Wu X. He W. Liu Z. Wu S. Chen C. (2014). DNA phosphorothioate modifications influence the global transcriptional response and protect DNA from double-stranded breaks. *Sci. Rep.* 4:6642. 10.1038/srep06642 25319634 PMC4198939

[B8] He W. Huang T. Tang Y. Liu Y. Wu X. Chen S. (2007). Regulation of DNA phosphorothioate modification in *Salmonella enterica* by DndB. *Sci. Rep.* 5:12368. 10.1038/srep12368 26190504 PMC4507180

[B9] He X. Ou H.-Y. Yu Q. Zhou X. Wu J. Liang J. (2015). Analysis of a genomic island housing genes for DNA S-modification system in *Streptomyces* *lividans* 66 and its counterparts in other distantly related bacteria. *Mol. Microbiol.* 65 1034–1048. 10.1111/j.1365-2958.2007.05846.x 17640271

[B10] Hu W. Wang C. Liang J. Zhang T. Hu Z. Wang Z. (2012). Structural insights into DndE from *Escherichia coli* B7A involved in DNA phosphorothioation modification. *Cell Res.* 22 1203–1206. 10.1038/cr.2012.66 22525332 PMC3391021

[B11] Ingram C. Brawner M. Youngman P. Westpheling J. (1989). *xylE* functions as an efficient reporter gene in *Streptomyces* spp.: use for the study of galP1, a catabolite-controlled promoter. *J. Bacteriol.* 171 6617–6624. 10.1128/jb.171.12.6617-6624.1989 2592344 PMC210555

[B12] Kieser T. Bibb M. J. Buttner M. J. Chater K. F. Hopwood D. A. (2000). *Practical Streptomyces Genetics.* Norwich: John Innes Foundation.

[B13] Liang J. Wang Z. He X. Li J. Zhou X. Deng Z. (2007). DNA modification by sulfur: analysis of the sequence recognition specificity surrounding the modification sites. *Nucleic Acids Res.* 35 2944–2954. 10.1093/nar/gkm176 17439960 PMC1888814

[B14] Ou H.-Y. He X. Shao Y. Tai C. Rajakumar K. Deng Z. (2009). dndDB: a database focused on phosphorothioation of the DNA backbone. *PLoS One* 4:e5132. 10.1371/journal.pone.0005132 19357771 PMC2663466

[B15] Saenger W. Hunter W. N. Kennard O. (1986). DNA conformation is determined by economics in the hydration of phosphate groups. *Nature* 324 385–388. 10.1038/324385a0 3785407

[B16] Schneider B. Neidle S. Berman H. M. (1997). Conformations of the sugar-phosphate backbone in helical DNA crystal structures. *Biopolymers* 42 113–124. 10.1002/(SICI)1097-0282(199707)42:1<113::AID-BIP10>3.0.CO;2-O 19350745

[B17] Svozil D. Kalina J. Omelka M. Schneider B. (2008). DNA conformations and their sequence preferences. *Nucleic Acids Res.* 36 3690–3706. 10.1093/nar/gkn260 18477633 PMC2441783

[B18] Tong T. Chen S. Wang L. Tang Y. Ryu J. Y. Jiang S. (2018). Occurrence, evolution, and functions of DNA phosphorothioate epigenetics in bacteria. *Proc. Natl. Acad. Sci. U.S.A.* 115 e2988–e2996. 10.1073/pnas.1721916115 29531068 PMC5879708

[B19] Vargason J. M. Eichman B. F. Ho P. S. (2000). The extended and eccentric E-DNA structure induced by cytosine methylation or bromination. *Nat. Struct. Biol.* 7 758–761. 10.1038/78985 10966645

[B20] Vargason J. M. Henderson K. Ho P. S. (2001). A crystallographic map of the transition from B-DNA to A-DNA. *Proc. Natl. Acad. Sci. U.S.A.* 98 7265–7270. 10.1073/pnas.121176898 11390969 PMC34657

[B21] Wang L. Chen S. Vergin K. L. Giovannoni S. J. Chan S. W. DeMott M. S. (2011). DNA phosphorothioation is widespread and quantized in bacterial genomes. *Proc. Natl. Acad. Sci. U.S.A.* 108 2963–2968. 10.1073/pnas.1017261108 21285367 PMC3041111

[B22] Wang L. Chen S. Xu T. Taghizadeh K. Wishnok J. S. Zhou X. (2007). Phosphorothioation of DNA in bacteria by *dnd* genes. *Nat. Chem. Biol.* 3 709–710. 10.1038/nchembio.2007.39 17934475

[B23] Xie X. Liang J. Pu T. Xu F. Yao F. Yang Y. (2012). Phosphorothioate DNA as an antioxidant in bacteria. *Nucleic Acids Res.* 40 9115–9124. 10.1093/nar/gks650 22772986 PMC3467049

[B24] Xu T. Liang J. Chen S. Wang L. He X. You D. (2009). DNA phosphorothioation in *Streptomyces* *lividans*: mutational analysis of the *dnd* locus. *BMC Microbiol.* 9:41. 10.1186/1471-2180-9-41 19232083 PMC2653506

[B25] Xu T. Yao F. Zhou X. Deng Z. You D. (2010). A novel host-specific restriction system associated with DNA backbone S-modification in *Salmonella*. *Nucleic Acids Res.* 38 7133–7141. 10.1093/nar/gkq610 20627870 PMC2978375

[B26] Yang Y. Xu G. Liang J. He Y. Xiong L. Li H. (2017). DNA backbone sulfur-modification expands microbial growth range under multiple stresses by its anti-oxidaton function. *Sci. Rep.* 7:3516. 10.1038/s41598-017-02445-1 28615635 PMC5471199

[B27] Yao F. Xu T. Zhou X. Deng Z. You D. (2009). Functional analysis of spfD gene involved in DNA phosphorothioation in *Pseudomonas* flurescens Pfo-1. *FEBS Lett.* 583 729–733. 10.1016/j.febslet.2009.01.029 19171139

[B28] You D. Wang L. Yao F. Zhou X. Deng Z. (2007). A novel DNA modification by sulfur: DndA is a NifS-like cysteine desulfurase capable of assembling DndC as an iron-sulfur cluster protein in *Streptomyces* *lividans*. *Biochemistry* 46 6126–6133. 10.1021/bi602615k 17469805

[B29] Zhang Y.-C. Liang J. Lian P. Han Y. Chen Y. Bai L. (2012). Theoretical study on steric effects of DNA phosphorothioation: b-helical destabilization in Rp-phosphorothioated DNA. *J. Phys. Chem. B* 116 10639–10648. 10.1021/jp302494b 22857608

[B30] Zhou X. He X. Liang J. Li A. Xu T. Kieser T. (2005). A novel DNA modification by sulphur. *Mol. Microbiol.* 57 1428–1438. 10.1111/j.1365-2958.2005.04764.x 16102010

