# Supplementary material for: Regulation of dndB Gene Expression in Streptomyces lividans
Source: Front Microbiol. 2018 Oct 8;9:2387. doi: 10.3389/fmicb.2018.02387 (PMC6186775; doi:10.3389/fmicb.2018.02387)
Supplement: Supplementary file 1 [file Table_1.DOC]

# Supplementary Materials

**Involvement of DNA sulfur modification in epigenetic regulation**

**Daofeng Dai^1^, Tianning Pu^2^, Jingdan Liang^2^, Zhijun Wang^2*†^ and Aifa Tang^1*†^**

^1^Health Science Center, The First Affiliated Hospital of Shenzhen University； Institute of Translational Medicine, Shenzhen Second People's Hospital, Shenzhen, China

^2^State Key Laboratory of Microbial Metabolism and School of Life Science and Biotechnology, Shanghai JiaoTong University, Shanghai, China

**^*^Correspondence:**

Zhijun Wang

wangzhijun@sjtu.edu.cn

Aifa Tang

tangaifa2018@email.szu.edu.cn

**^†^**These authors have contributed equally to this work.

**Table S1. Strains and plasmids used in the study**

| **Strains and plasmids** | **Description** | **Source/reference** |
| --- | --- | --- |
| **Strains** |  |  |
| *S. lividans* 1326 | Wild type, *dnd^+^*, SLP2+, SLP3+ | [1] |
| HXY1 | *dndA^-^*, *dndA* deletion mutant | [2] |
| HXY2 | *dndB^-^*, *dndB* in-frame deletion mutant | [3] |
| HXY3 | *dndC^-^*, *dndC*  deletion mutant | [2] |
| HXY6 | *dnd^-^*, the whole *dnd* gene cluster deletion mutant | [2] |
| *E. coli* DH5α | F^-^, *recA*, *lacZ*, ∆M15 | [4] |
| *E. coli* ET12567  /pUZ8002 | pUZ8002, *recF*, *dam*, *dcm*, *hsds*, *cml^r^*, *km^r^* | [5] |
| **Plasmids** |  |  |
| pSET152 | *aac(3)*, *lacZ*, *rep^pUC^*, *att^ΦC31^*, *oriT* | [6] |
| pIJ4083 | *xylE*, *tsr*, *ori^pIJ101^* | [7] |
| pJTU3700 | Insertion of *xylE* from pIJ4083 into pSET152 | [8] |
| pJTU3707 | Insertion of P*dnd*B into pJTU3700 | [8] |
| pMD18-T | Vector for DNA sequencing, *bla* | Takara |

**References**

1.Zhou, X. et al. *Streptomyces coelicolor* A3(2) lacks a genomic island present in the chromosome of *Streptomyces lividans* 66. *Appl. Environ. Microbiol*. 70, 7110-8 (2004).

2.He X, et al. Analysis of a genomic island housing genes for DNA S-modification system in *Streptomyces lividans* 66 and its counterparts in other distantly related bacteria. *Mol. Microbiol.*, 65, 1034-1048 (2007).

3. Liang, J. et al. DNA modification by sulfur: analysis of the sequence recognition specificity surrounding the modification sites. *Nucleic Acids Res.* 35, 2944–2954 (2007)

4. Hanahan, D. Echerichia coli with plasmids. *J. Mol. Biol.* 166, 557-580 (1983).

5. Flett, F. et al. High efficiency intergeneric conjugal transfer of plasmid DNA from *Echerichia coli* to methyl DNA-restricting streptomycetes. *FEMS Microbiol. Lett*. 155, 223-9 (1997).

6. Bierman, M. et al. Plasmid cloning vectors for the conjugal transfer of DNA from *Echerichia coli* to *Streptomyces* spp. *Gene* 166, 43-9 (1992).

7. Timothy, C. et al. *Streptomyces* promoter-probe plasmids that utilise the *xylE* gene of *Pseudomonas putida. Nucleic Acids Res*. 18, 1077 (1989).

8.Dai D, et al. DNA Phosphorothioate Modification Plays a Role in Peroxides Resistance in *Streptomyces lividans. Front. Microbiol.* 7, 1380 (2016).
