# Supplementary material for: Regulation of dndB Gene Expression in Streptomyces lividans
Source: Front Microbiol. 2018 Oct 8;9:2387. doi: 10.3389/fmicb.2018.02387 (PMC6186775; doi:10.3389/fmicb.2018.02387)
Supplement: Supplementary file 2 [file Table_2.DOCX]

# Supplementary Materials

**Involvement of DNA sulfur modification in epigenetic regulation**

**Daofeng Dai^1^, Tianning Pu^2^, Jingdan Liang^2^, Zhijun Wang^2*†^ and Aifa Tang^1*†^**

^1^Health Science Center, The First Affiliated Hospital of Shenzhen University； Institute of Translational Medicine, Shenzhen Second People's Hospital, Shenzhen, China

^2^State Key Laboratory of Microbial Metabolism and School of Life Science and Biotechnology, Shanghai JiaoTong University, Shanghai, China

**^*^Correspondence:**

Zhijun Wang

wangzhijun@sjtu.edu.cn

Aifa Tang

tangaifa2018@email.szu.edu.cn

**^†^**These authors have contributed equally to this work.

**Table S2. Primers used in this study**

| **Primer and purpose** | **sequence** |
| --- | --- |
| **For mutagenesis** |  |
| R1F | AACTATTTACCTGACGTACGA |
| R1R | CGTGGGACGTGCAGATTAACGC |
| R2F | AATCCGATAGCGTGAGCGGGT |
| R2R | CGTGGTAAATGACGTACGGGA |
| S9F | ACCGGTGCGCTCGCCGTAGGG |
| S9R | CGGCGCCACATCATCACCTCC |
| S12F | ACCGGCCAGATCGTGATCTCG |
| S12R | CCGCATCCCTACGCCGCTGTC |
| **For Southern blotting** |  |
| probe1F | CGCTCGTCGTCCATCTCCTC |
| probe1R | CAAGTCGGCGAACATCTGCT |
| Marker447F | TACGATAGCGTGAGCGGGTG |
| Marker447R | GGTCGGGGTTCTCCTTCAGC |
| Marker830F | CATGTCGATGGGGGTAGTGA |
| Marker830R | TCGGGTGCCAGCTCGTCCTC |
| Marker1266F | GTGAGGTCGCCGGGTACCTT |
| Marker1266R | CCACAAAGCCCTTGAACACT |
| probe2F | TCAGGTCAGCACGGTCATGAA |
| probe2R | CGACTTCTGCCGGTTTTGAGT |
| marker500F | TCAGGTCAGCACGGTCATGAA |
| marker500R | ATCTGAAAGGTATGGCGGCTG |
| marker1018F | CAGGTCAGCACGGTCATGAATCG |
| marker1018R | TGGCTGCCCGATTCCTGATCA |
| marker1428F | TCAGGTCAGCACGGTCATGAA |
| marker1428R | TGAGCGTTAATCTGCACGTCC |
| **For quantitative real-time PCR** | |
| rrnAF | AGTAACACGTGGGCAACTGC |
| rrnAR | CTCAGACCAGTGTGGCCGGT |
| dndCF | GCAACCTCCCCAACTCCCTT |
| **Primer and purpose** | **sequence** |
| dndCR | CTTGTTCTTGTGGCCCCACG |
| dndDF | CTATGGGGGCTGGCTAAAGT |
| dndDR | AGTACCGGTCGACGAGGTGT |
| dndEF | GCCTGACCCAGTGGAACGAA |
| dndER | GGTAGATGTCACCGTGCGGG |
